# DEAD-Box Helicase 27 Triggers Epithelial to Mesenchymal Transition by Regulating Alternative Splicing of Lipoma-Preferred Partner in Gastric Cancer Metastasis

**DOI:** 10.3389/fgene.2022.836199

**Published:** 2022-05-04

**Authors:** Yirong Jin, Suzhen Yang, Xiaoliang Gao, Di Chen, Tingting Luo, Song Su, Yanting Shi, Gang Yang, Lei Dong, Jie Liang

**Affiliations:** ^1^ State Key Laboratory of Cancer Biology, National Clinical Research Center for Digestive Diseases, Air Force Military Medical University, Xi’an, China; ^2^ Department of Digestive Disease and Gastrointestinal Motility Research Room, Xi’an Jiaotong University, Xi’an, China; ^3^ Key Laboratory of Resource Biology and Biotechnology in Western China, Ministry of Education, School of Medicine, Northwest University, Xi’an, China

**Keywords:** DDX27, gastric cancer metastasis, LPP, alternative splicing, EMT

## Abstract

DEAD-box helicase 27 (DDX27) was previously identified as an important mediator during carcinogenesis, while its role in gastric cancer (GC) is not yet fully elucidated. Here, we aimed to investigate the mechanism and clinical significance of DDX27 in GC. Public datasets were analyzed to determine DDX27 expression profiling. The qRT-PCR, Western blot, and immunohistochemistry analyses were employed to investigate the DDX27 expression in GC cell lines and clinical samples. The role of DDX27 in GC metastasis was explored *in vitro* and *in vivo*. Mass spectrometry, RNA-seq, and alternative splicing analysis were conducted to demonstrate the DDX27-mediated molecular mechanisms in GC. We discovered that DDX27 was highly expressed in GCs, and a high level of DDX27 indicated poor prognosis. An increased DDX27 expression could promote GC metastasis, while DDX27 knockdown impaired GC aggressiveness. Mechanically, the LLP expression was significantly altered after DDX27 downregulation, and further results indicated that LPP may be regulated by DDX27 *via* alternative splicing. In summary, our study indicated that DDX27 contributed to GC malignant progression *via* a prometastatic DDX27/LPP/EMT regulatory axis.

## Introduction

Gastric cancer was responsible for over 1 million new cases and 0.7 million deaths in 2020, which is still the main cause of cancer morbidity and mortality worldwide (Sung et al., 2021). Despite advances in early diagnosis and treatment, the incidence of recurrent and metastatic gastric cancer remains stubbornly high (Uemura et al., 2001). Metastasis is one of the most significant features of advanced GC, and a large proportion of GC patients in advanced stages benefit less than expected mainly due to metastasis (Soerjomataram and Bray 2021). GC is a prevalent but heterogeneous disease, arising from genetic accumulation and epigenetic alterations (Tan and Yeoh 2015). This gradual accumulation of gene alterations leads to continuous growth and metastatic advantage of neoplastic cells and substantially promotes GC malignant progression (Hayakawa et al., 2021). Thus, it is an urgent necessity to identify specific targets contributing to GC metastasis.

DDX27 belongs to the DEAD-box RNA helicases family, which has been verified to participate in carcinogenesis (Mazurek et al., 2012; Zhang et al., 2019; Mo et al., 2021). Accumulative evidence suggests that DDX27 serves as an oncogene in various cancers, such as colorectal cancer (CRC), hepatocellular carcinoma (HCC), and GC. For example, DDX27 accelerates CRC progression by forming a DDX27-NPM1-NFκB functional axis (Tang et al., 2018). Also, Tsukamoto et al. reported that DDX27 accelerated GC proliferation by inducing TP53-dependent cell cycle arrest (Tsukamoto et al., 2015). Meanwhile, DDX27 causes chemotherapeutic resistance toward epirubicin and cisplatin in the GC cells (Zhou et al., 2015). Additionally, DDX27 clues the unfavorable outcomes of HCC patients (Wang et al., 2015). Nevertheless, the role of DDX27 in malignant tumors still remains largely unclear, especially in the GC metastatic process.

Alternative splicing (AS) plays an indispensable role in enlarging gene expression patterns and enriching protein diversity. Dysregulation of splicing variants has always been strongly associated with tumor malignancy, including poor differentiation, metastasis, and poor prognosis of patients (Hönig et al., 2002; Dardenne et al., 2012; Germann et al., 2012). Abnormal expression levels and activities of splicing factors linked with abnormal AS patterns were extensively detected in different kinds of tumors (Rahman et al., 2020). A study using 32 different types of cancer tissues revealed that abnormal AS events always happen during cancer progression (Kahles et al., 2018). Meanwhile, the DDX family is generally involved in spliceosome assemblies and functioned as one of the canonical regulators of splicing in RNA metabolism (Ameur et al., 2020), which is gaining growing attention in cancer research for its vital roles in AS of many tumor-associated genes, such as macroH2A1 histones (NFAT5) (Germann et al., 2012) and CD44 (Hönig et al., 2002).

Lipoma-preferred partner (LPP) is located at chromosome 3q27-q28, owned by the zyxin family of proteins (Ngan et al., 2018). It is primarily expressed in the cell periphery of focal adhesion, thus participating in cytoskeletal organization, cell motion, and mechanosensing (Grunewald et al., 2009). The malignant role of LPP within carcinogenesis has been previously revealed. For example, LPP mediates breast cancer cells metastasize to lungs by participating in the formation of invadopodia and regulating cell motility. LPP promotes tumor angiogenesis and confers chemoresistance to ovarian cancer (Leung et al., 2018). In addition, LPP has been noticed to be regulated in an alternative splicing manner (Petit et al., 1996), which contains a number of transcription variants (Yamanaka et al., 2008).

Here, we elucidated the critical role of DDX27 in gastric cancer progression and found that DDX27 promoted the GC EMT process by regulating the alternative splicing of LPP.

## Materials and Methods

### Public Datasets

Public datasets were obtained from the Gene Expression Omnibus (GEO) database, the Cancer Genome Atlas (TCGA) program, and Gene Expression Profiling Interactive Analysis 2 (GEPIA2). Each dataset was applied for separate usage, according to the data characteristics respectively. In details, gastric cancer transcriptome data are fully or partly acquired from the data generated by TCGA Research Network: https://www.cancer.gov/tcga. GEPIA2 (http://gepia2.cancer-pku.cn/) was performed to analyze the difference of DDX27 between gastrointestinal tumors and healthy specimens at translation levels. The cBioPortal (https://www.cbioportal.org/) database and the gastric cancer dataset are employed to analyze the mutation information of DDX27 and clinical characteristics in gastric cancer. The effects of DDX27 and LPP expression on the survival time among the GC patients were analyzed by the Kaplan–Meier plotter (http://kmplot.com/analysis/).

### Patients and Specimens

A total of 104 GC specimens (52 paired primary tumor and normal tissues) were obtained from Xijing hospital, Air Force Military University, between the year of 2018 and 2020, in accordance with ethical approvals. Fresh tissue specimens were divided into three parts: the first part is formalin-fixed, the second is paraffin-embedded, and the last was kept at liquid nitrogen. The study was approved by the Clinical Research Ethics Committee of the Air Force Military Medical University and Xijing Hospital. Informed consent was obtained from all the subjects involved in the study.

### Cell Culture

All the cell lines (MKN-28, AGS, BGC-823, SNU-1, MKN-45, HGC-27, and GES-1) were restored in our laboratory for research. DMEM (Gibco, Grand Island, NY, United States) was used to culture HGC-27 only, while the others were cultured in RPMI1640 (Gibco). For the process, 10% fetal bovine serum (FBS, Gibco, Grand Island, NY, United States), 100 μg/ml streptomycin, and 100 IU/ml penicillin were added into a medium to culture cells at 37°C in a humidified 5% CO_2_ atmosphere.

### Quantitative Reverse Transcription–Polymerase Chain Reaction

Following the instructions of the manufacturer, the total RNA in cell lines and tissues was extracted using the RNAiso reagent (TaKaRa, Japan). PrimeScript RT Master Mix (TaKaRa) was selected to perform reverse transcription. Synthesized cDNA was used to conduct real-time PCR by the SYBR Premix Ex Taq II (TaKaRa). The CFX96 RealTime PCR detection system (Bio-Rad, CA, United States) was adopted for detection. The determined relative expression levels of target genes were obtained using the 2^−ΔΔCt^ method, and β-actin was chosen as the internal control for mRNAs. The primers used are listed in Supplementary Table S1.

### Western Blot

The total proteins from cell or tissue lysates were transferred onto the nitrocellulose membranes after being resolved by SDS-PAGE. The membranes were blocked in 5% fat-free milk and then incubated with primary specific antibodies at 4°C overnight (DDX27: ab177938, Abcam; LPP: #3389, CST, MA, United States; E-cadherin: #14472, CST; Vimentin: #5741, CST; N-cadherin: #13116, CST; ZO-1: #13663, CST; β-actin: #3700, CST). Appropriate HRP-conjugated secondary antibody was used to further incubate the protein band for 1 h. The Molecular Imager ChemiDoc XRS + Imaging System was used for membrane visualization, and protein quantification was obtained by Image Lab software.

### Immunohistochemistry and Evaluation

IHC staining was performed on paraffin sections or microarrays. In brief, all the slides were dewaxed at 65°C for 2 h, and each slide got antigen repairment with ethylenediaminetetraacetic acid (EDTA) or citric acid. Hydrogen peroxide was used to eliminate endogenous peroxidase (EGPO). After blocking by using serum for 1 h, the slides were incubated with specific primary antibodies. At last, the sections were incubated with horseradish peroxidase (HRP)-labeled secondary antibody, followed by coloring with diaminobenzidine (Dako). All the slides were counterstained with hematoxylin.

The IHC protein expression level was evaluated by two independent pathologists using semi-quantitative scoring of the intensity methods. The scoring system contains two parameters, including the percentage of positive cells and the intensity of staining. The percentage of cells was divided into four degrees: < 1% (1), 1–25% (2), 26–75% (3), and >75% (4). Negative (0), weak (1), moderate (2), and strong (3) were conducted to evaluate the intensity of staining. The product of the two parameters was equaled to get a final score. The final score 0–6 was graded as low, and 8–12 was defined as high, which represented “low expression” and “high expression” of the target molecules.

### Stable Cell Lines Construction

To silence the target genes, the siRNA hairpin sequence was cloned into the GV248-Puro lentivirus vector, and a fragment of cDNA was cloned into the GV492-Puro lentivirus vector to overexpress the target gene. Corresponding negative controls (LV-Control and shControl) were also constructed by Shanghai GeneChem Company. Hitrans G (GeneChem, China) was applied for cell infection, following the instruction. In brief, the cells were cultured in OPTI-MEM (Gibco, United States) and transfected with lentivirus for 24 h; then, the medium was replaced with fresh complete media containing puromycin (Merck Millipore, Germany) for stable cell selection for at least 1 week. The sequences used are exhibited in Supplementary Table S1. The efficiency of lentivirus was detected using the real-time PCR and Western blot.

### Trans-Well Assay

Corning chambers containing the 8-μm pore polycarbonate membrane filter were selected to perform the trans-well migration assays. The 8 × 10^4^ cells were added to the upper chamber after being suspended with a serum-free medium. A measure of 600 μl of culture medium containing 20% FBS was added into the bottom chamber. The cells on top of the membrane were wiped off after 24 h of incubation. The cells migrated to the bottom surface of the filter, were fixed, and then stained with 1% crystal violet. Each trans-well insert was microscopically imaged in five randomly selected regions at x20 in triplicates. The cells that migrated to the bottom surface were shot for statistical analysis. Similarly, invasion assays were performed, following the same protocol as migration assays, except that the 8-μm microporous filter was coated with the configured Matrigel (Corning) to examine the cell invasive abilities.

### Wound Healing

The cells were seeded in 6-well plates. When grew to 90% confluence, the attached cell layers were carefully scratched by a 200-μl pipette tip to generate wounds. The remaining adherent cells were incubated with the serum-free medium and then photographed at different time points (0, 24 and 48 h) using the inverted microscope (Olympus, Tokyo, Japan).

### Metastasis Assays *In Vivo*

BALB/c nude mice were raised in specific comfortable and pathogen-free conditions. The mice (ten, 4–6-week mice per group, 18–22 g) were injected with cells transferred with recombinant lentiviruses *via* the tail vein to construct the vivo metastatic model. Separated metastatic nodes in lung specimens were separated from the sacrificed mice and fixed with paraformaldehyde (1%) about 6–8 weeks later. After H&E staining, the nodes were counted to evaluate cell metastasis potentials. The animal study was reviewed and approved by the Institutional Animal Care and Use Committee of the Air Force Military Medical University.

### Gene Set Enrichment Analysis

GSEA was performed by GSEA 4.1.0 software (GSEA, United States), and the gene set database was set as ftp.broadinstitute.org://pub/gsea/gene_sets/h.all.v7.4.symbols. The number of permutations was set at 1000 and the permutation type as phenotype. For single-gene GSEA, TCGA rpkm data were divided into two groups. The top 25% was identified as high expression, and the bottom 75% was perceived as low expression, followed by GSEA analysis.

### Transcriptome Sequencing and Alternative Splicing Analysis

RNAseq was conducted between the DDX27 knockdown and control cell lines. The transcriptome sequencing was performed on the Illumina sequencing platform. Illumina PE libraries (∼300bp) were constructed for sequencing, and the sequencing data obtained were quality controlled, after which the transcriptome data were analyzed using bioinformatics tools. The clean data are compared with the reference genome to obtain the whole transcriptomic information. RPKM (Reads per Kilobase per Million Reads) values as a measure of the gene expression. For the AS analysis, we used rMATS (replicate Multivariate Analysis of Transcript Splicing, University of California) software for the variable splicing analysis of the differences. I is the exon inclusion reads, and S is the exon skipping reads, LI is the effective length of the inclusion isoform (length of the inclusion form), and LS is the effective length of the skipping isoform (length of the skipping form); then, the estimated value of ψ is

$$\hat{\psi }=\frac{I/L_{I}}{I/L_{I}+S/Ls}.$$

The alternative splicing threshold is set as *p* value < 0.05, difference |Δψ|>0.1. GO term annotation and KEGG pathway associated with alternative splicing are analyzed with *p* < 0.05.

### Statistical Analysis

All data were expressed as the mean ± standard errors. GraphPad Prism 8.4.0 software (GraphPad Software, United States) and SPSS software (IBM SPSS, Armonk, NY, United States) were employed for the analysis. Considering the data characteristics, differential analysis was performed by the Wilcoxon rank-sum test, two-group t-test, ANOVA, and Student’s t test. The cut-off value for the high or low expression was defined with ROC analysis. The Cox regression model, Kaplan–Meier curve, and log-rank test were applied for the survival analysis. Spearman correlation analysis was utilized for correlation assessment. *p* < 0.05 was considered statistically significant.

## Results

### DEAD-Box Helicase 27 Exhibits an Elevated Expression Profile in Gastric Cancer

**FIGURE 1:**
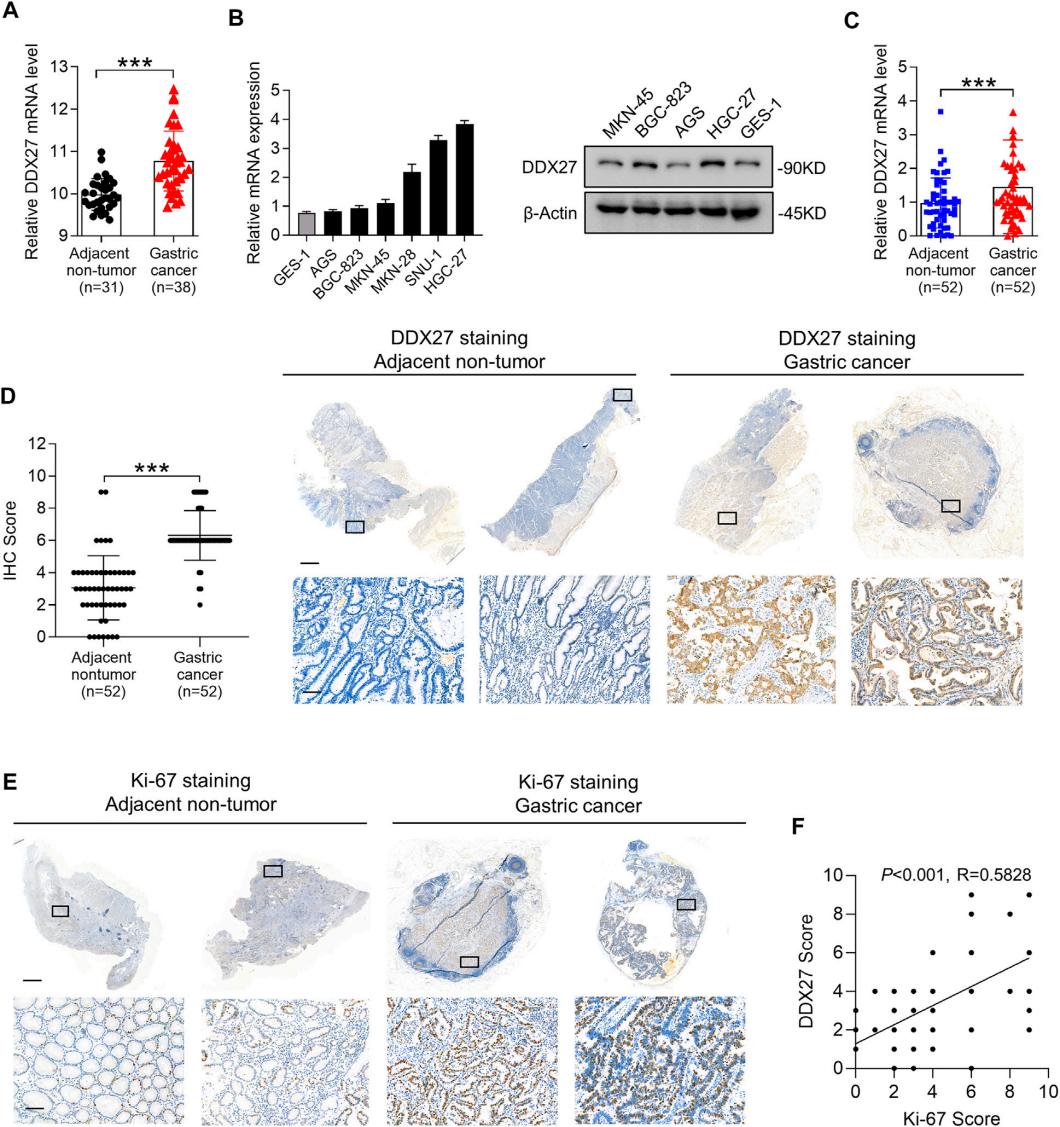
DDX27 exhibits an elevated expression profile in GC. **(A)** DDX27 transcript levels between the adjacent nontumor and GC tissues in the GEO database (GSE13911, ***p < 0.001, by Student’s t test). **(B)** Quantitative reverse transcription PCR (qRT-PCR) (left) and Western blot (right) analyses of the DDX27 expression in the normal gastric epithelial cell line (GES-1) and gastric cancer cell lines (ASG, BGC-823, MKN-45, MKN-28, SNU-1, and HGC-27). **(C)** DDX27 expression was analyzed by qRT-PCR in paired surgically resected GC (*n* = 52) and adjacent nontumor tissues (*n* = 52) (***p < 0.001, by Student’s t test). **(D)** IHC score of DDX27 in clinically paired collected adjacent nontumor (*n* = 52) and GC tissues (*n* = 52) (left, ***p < 0.001, by Student’s t test)). Representative images of staining were exhibited in the right. Scale bars: 1000 μm (up) or 100 μm (below). **(E)** IHC staining images of Ki-67 in the adjacent nontumor (*n* = 52) and GC tissues (*n* = 52). Scale bars:1000 μm (up) or 100 μm (below). **(F)** Correlation analysis between DDX27 and the Ki-67 IHC staining score. (*p* < 0.001, *R* = 0.5828, by Pearson correlation method). *n* ≥ 3, the data are presented as the mean ± standard deviation (SD). A high DDX27 expression indicates poor clinical prognosis.

Large amounts of studies have regarded DDX27 as vital cancer-promoting genes in distinct tumors (Tsukamoto et al., 2015; Tang et al., 2018). However, the definite role of DDX27 in GC remains largely uncharacterized. Therefore, we first assessed the DDX27 expression among various gastrointestinal tumors. TCGA gene expression profiling showed that the high expression of DDX27 was exhibited in the gastrointestinal tumor tissues, especially in stomach adenocarcinoma (STAD) (Supplementary Figure S1A). From the Gene Expression Omnibus (GEO) database, we again found that GC tissues contained a higher level of DDX27 than the adjacent nontumor tissues (Figure 1A).

Subsequently, we detected the DDX27 mRNA and protein levels in different GC cell lines and noticed that compared to the normal gastric epithelial cell line (GES-1), DDX27 was significantly upregulated in the GC cells (AGS, BGC-823, MKN-45, MKN-28, SNU-1, and HGC-27) (Figure 1B). By analyzing the paired clinical collected GC and adjacent nontumor tissues with qRT-PCR, an elevated mRNA level of DDX27 was found in the GC tissues rather than in nontumor tissues (Figure 1C, *n* = 52).

Ki-67 is a representative marker of cancer malignancy, and the paired clinical specimens were utilized to observe the expression of DDX27 and Ki67 with IHC analysis. IHC staining indicated that DDX27 and Ki-67 were upregulated in the GC tissues and mainly distributed in the nucleus (Figures 1D,E). In addition, the DDX27 IHC score exhibited a positive correlation with the Ki67 IHC score in the GC tissues (Figure 1F). Overall, we can draw the conclusion that DDX27 was significantly upregulated in GC and may participate in facilitating the GC malignancy development.

### High DEAD-Box Helicase 27 Expression Indicates Poor Clinical Prognosis

**FIGURE 2:**
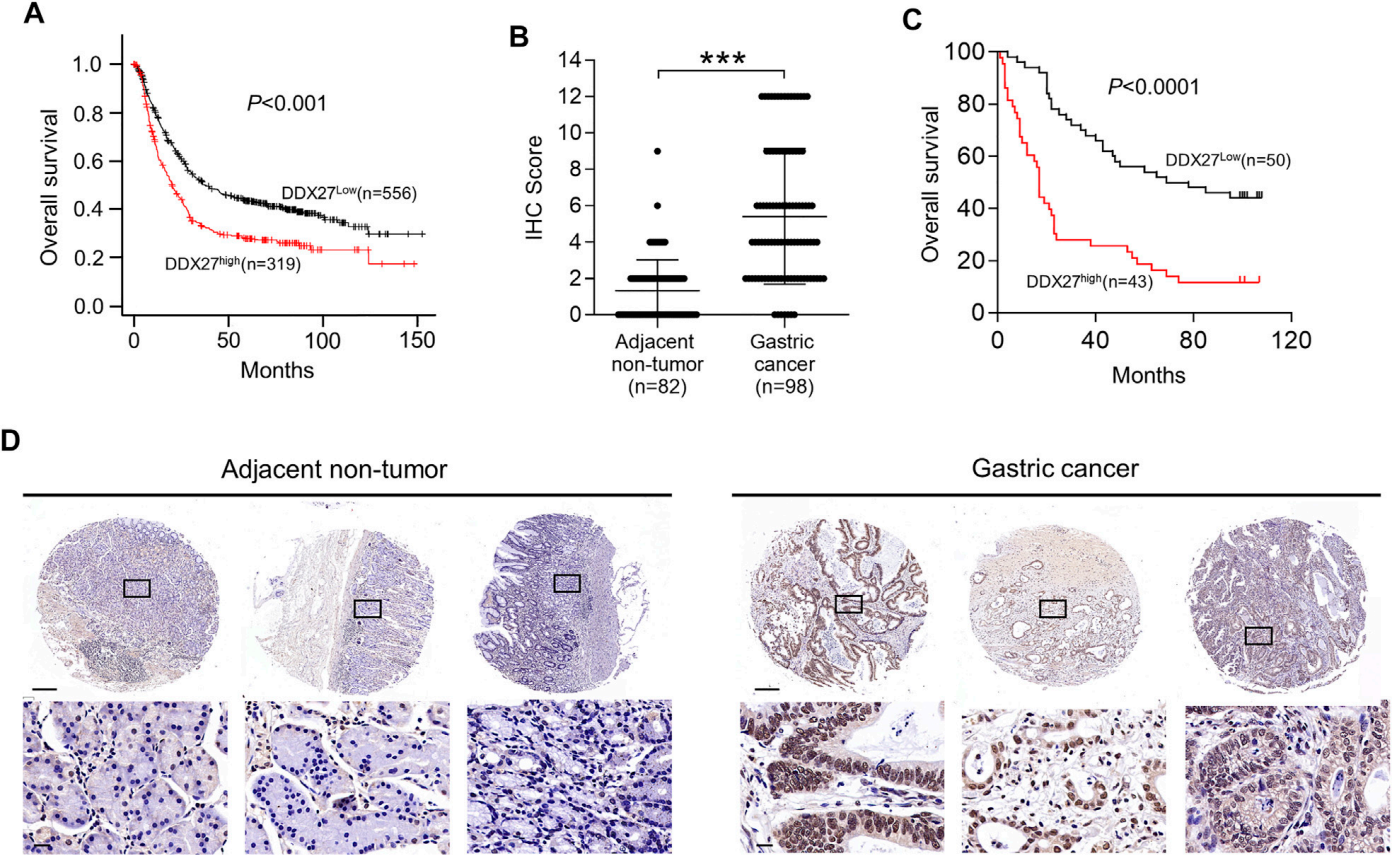
High DDX27 expression indicated a poor clinical prognosis. **(A)** Kaplan-Meier analysis of overall survival (OS) between GC patients with high (*n* = 319) or low (*n* = 556) DDX27 expression from the dataset. (*p* < 0.001, by log-rank test). **(B,D)** IHC scoring of paired adjacent nontumor (*n* = 82) and GC tissues (*n* = 98) [**(B)**, ***p < 0.001, by Student’s t-test] with the representative images of IHC staining for DDX27 protein levels **(D)**. Scale bars: 250 μm (up) or 20 μm (below). **(C)** Kaplan–Meier analysis was used to evaluate the DDX27 expression in patients with GC form tissue microarray (*p* < 0.001, by log-rank test). *n* ≥ 3, the data are presented as the mean ± SD. DDX27 is a critical mediator of GC metastasis *via* EMT.

To explore the clinical signature of DDX27 in GC, we conducted Kaplan–Meier (KM) analysis and discovered GC patients with a higher DDX27 expression always had worse overall survival (OS) (*p* < 0.001; Figure 2A). IHC staining of tissue microarray containing GC (*n* = 98) exhibited a relatively high expression level of DDX27 in GC compared with the nontumor tissues (*n* = 82) (*p* < 0.001; Figures 2B,D).

In line with the results of Figure 2A, IHC staining of the tissue microarray showed that GC patients with a high DDX27 expression tend to have a worse prognosis (*p* < 0.0001; Figure 2C). Moreover, DDX27 had a positive correlation with the depth of invasion, lymph node metastasis, and distant organ metastasis (*p* < 0.05, Table 1). Univariate analysis revealed that tumor size, invasion depth, lymph node metastasis, distant organic invasion, AJCC, and DDX27 expression all were associated with OS (*p* < 0.05, Table 2), and multivariate Cox regression analysis validated that a higher DDX27 expression was an independent risk factor for the GC patient prognosis and could predict patient’s shorter OS (*p* < 0.05, Table 3).

**TABLE 1:** Association between DDX27 and clinicopathological parameters of GC patients.

Characteristic	Total	DDX27		*p*
		Low	High	
Gender				
Female	36	20	16	0.823447
Male	62	33	29	
Age (years)				
≤60	35	21	14	0.380858
>60	63	32	31	
Location				
Cardia	11	4	7	0.648887
Fundus/body of stomach	29	17	12	
Antrum	50	27	23	
Diffuse	7	4	3	
Tumor size				
≤6 cm	61	36	25	0.126739
>6 cm	30	15	20	
Depth of invasion				
T 1–3	77	49	28	0.000479
T 4	20	4	16	
Metastases to other organs				
M 0	89	51	38	0.044156
M 1	9	2	7	
Number of positive lymph nodes				
≤5	57	36	21	0.043427
>5	38	16	22	

PS: bold fonts represent *p* < 0.05.

**TABLE 2:** Prognostic factors in patients with gastric cancer by univariate analysis.

Characteristic	*n*	HR	95% CI	*p*
Gender				
Female	35	1.223855	0.748886–2.000066	0.420199
Male	58			
Age (years)				
≤60	32	1.05675	0.6329–1.7643997	0.832845
>60	61			
Location				
Cardia	10	0.905378	0.647746–1.265480	0.560687
Fundus/body of stomach	26			
Antrum	49			
Diffuse	7			
Tumor size				
≤6 cm	61	2.09	1.2619–3.46166	0.004190
>6 cm	30			
Depth of invasion				
T 1–3	73	1.9813	1.13713–3.4523	0.015797
T 4	19			
Metastases to other organs				
M 0	85	3.948667	1.8432–8.58917	0.000436
M 1	8			
Number of positive lymph nodes				
≤5	54	2.7105	1.6359–4.49099	0.000109
>5	36			
AJCC				
1/2	39	2.069	1.24078–3.450429	0.005323
3/4	53			
DDX27				
Low	50	3.085071	1.877780–5.068573	0.000009
High	43			

PS: bold fonts represent *p* < 0.05.

**TABLE 3:** Multivariate analysis using the Cox proportional hazards model.

Characteristic	*n*	HR	95% CI	*p*
Tumor size				
≤6 cm	61	1.982423	1.143308–3.437395	0.014815
>6 cm	30			
Depth of invasion				
T 1–3	73	1.169362	0.619943–2.205699	0.628930
T 4	19			
Metastases to other organs				
M 0	85	1.648962	0.682601–3.983401	0.266385
M 1	8			
Number of positive lymph nodes				
≤5	54	2.456772	1.223911–4.931509	0.011462
>5	36			
AJCC				
1/2	39	0.796997	0.396643–1.601450	0.523923
3/4	53			
DDX27				
Low	50	2.561683	1.437016–4.566559	0.001427
High	43			

PS: bold fonts represent *p* < 0.05.

In addition, we investigated the DDX27 genetic alterations among a GC cohort (OncoSG, 2018) with whole-genome sequencing and found that DDX27 amplification account for about 21%, indicating a high frequency of amplification of DDX27 among the clinical GC patients. Specific clinical features of DDX27 amplification are shown in Supplementary Figure S1B. All these results suggested that DDX27 have the potential of being a potential indicator in GC prognosis.

### DEAD-Box Helicase 27 is a Critical Mediator of Gastric Cancer Metastasis *via* Epithelial Mesenchymal Transition

**FIGURE 3:**
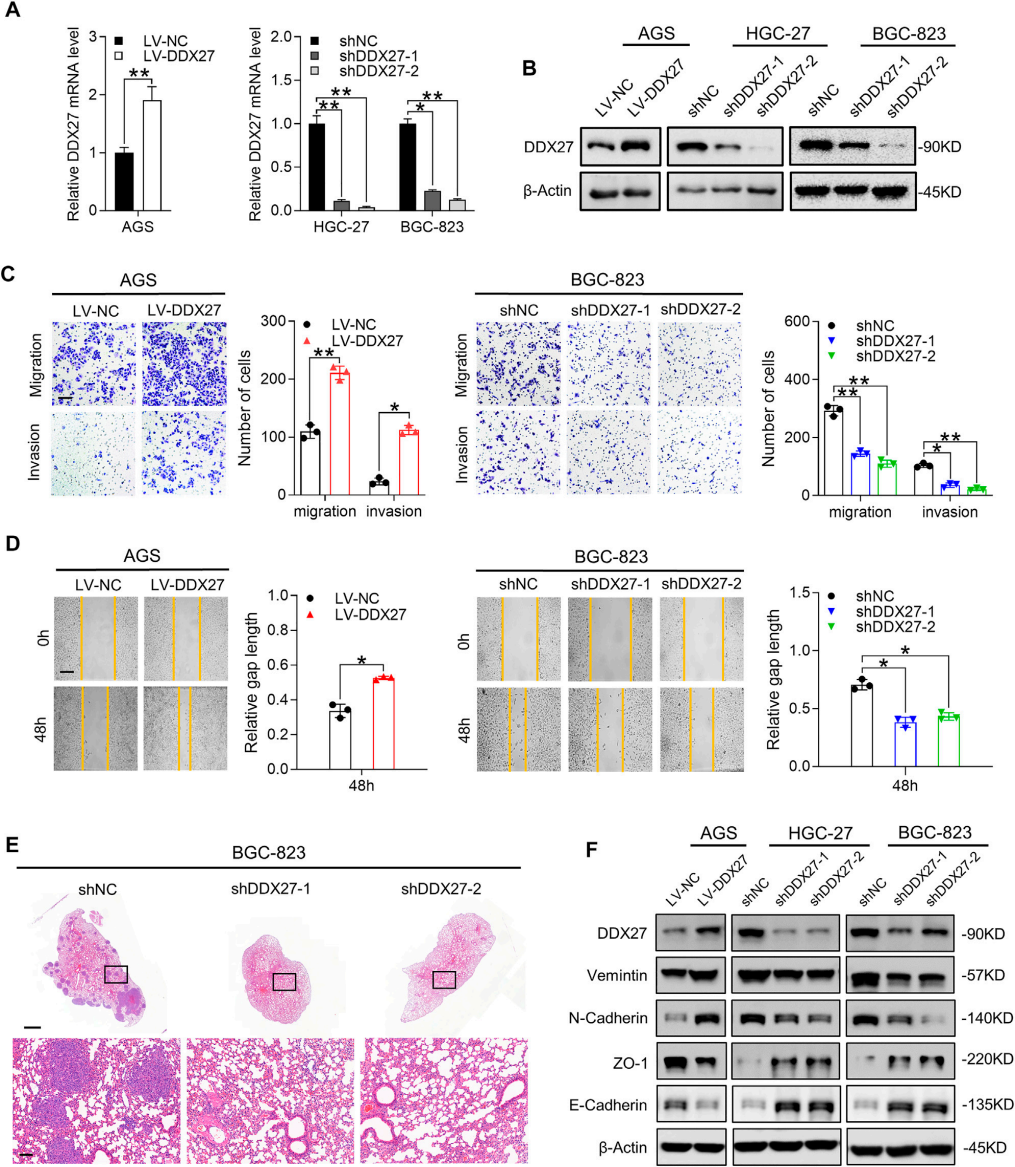
DDX27 is a critical mediator of GC metastasis *via* EMT. **(A,B)** Transcript level **(A)** and protein expression **(B)** of DDX27 in DDX27-knockdown and overexpressed cell models (**p < 0.01, *p < 0.05, by ANOVA). **(C)** Metastatic ability assessed by trans-well assay for DDX27-overexpressed and -knockdown cell models (left, overexpression vs. control; right, knockdown vs. control). Scale bars: 12.5 μm (**p < 0.01, *p < 0.05, by ANOVA). **(D)** Wound healing and statistical analysis for indicated cells (left, overexpression vs. control; right, knockdown vs. control) Scale bars: 25 μm. (*p < 0.05, by ANOVA). **(E)** Hematoxylin-eosin **(H,E)** staining for metastatic nodules in the dissected lung specimen from nude mice (10 mice in each group). Scale bars:1,000 μm (up) or 100 μm (below). **(F)** Protein level of EMT markers (E-cadherin, N-cadherin, vimentin, and ZO-1) among the indicated GC cells. *n* ≥ 3, the data are presented as mean ± SD. LPP is indispensable for DDX27-mediated GC migration and invasion.

The findings we have gained suggested us that the biological function of DDX27 may promote GC progression. To unveil the role of DDX27 in tumor malignancy, we successfully constructed a stable DDX27 overexpression (LV-DDX27) and knockdown (shDDX27) cell models in three GC cell lines (AGS, HGC-27, and BGC823) using recombinant lentivirus infection. The transfection efficiency is shown in Figures 3A,B.

Metastasis has been verified as a prototypical feature in tumor progression and a core hallmark of malignant behavior (Lambert et al., 2017). Therefore, we performed functional assays to fully characterize the effects of DDX27 during GC metastasis. Just as Tsukamoto et al. mentioned previously, DDX27 may contribute to GC invasiveness, yet they did not explore further (Tsukamoto et al., 2015). Our *in vitro* assay results confirmed their assumptions that overexpressed DDX27 could promote GC metastasis (Figures 3C,D left), while DDX27 inhibition substantially impaired GC cell motility (Figures 3C,D right and Supplementary Figure S1C). In *in vivo* lung metastatic models, when DDX27 was downregulated, the incidence of *in vivo* lung metastasis was significantly reduced (Figures 3E, Supplementary Figure S1D).

EMT is considered to be one of the most obvious traits in tumor metastasis, while N-cadherin upregulation and E-cadherin downregulation are typical indicators of EMT progress (Dongre and Weinberg 2019; Lambert and Weinberg 2021). Thus, we detected some typical EMT markers in DDX27 stable up- or downregulated cell lines and found that DDX27 could increase the protein levels of vimentin and N-cadherin and reduce ZO-1 and E-cadherin expressions in the GC cells (Figure 2F). To further illustrate the function of DDX27 in the EMT process, the Gene Set Enrichment Analysis (GSEA) was proceeded, and the results suggested that EMT was one of the most impaired pathways in the DDX27-deficient GC cells compared with the controls (Supplementary Figure S1E).

In this part, we determined that DDX27 could strengthen the metastatic capacity of the GC cells, and DDX27 may realize its promotive effects by triggering the EMT process in GC.

### Lipoma-Preferred Partner is Indispensable for DEAD-Box Helicase 27–Mediated Gastric Cancer Migration and Invasion

**FIGURE 4:**
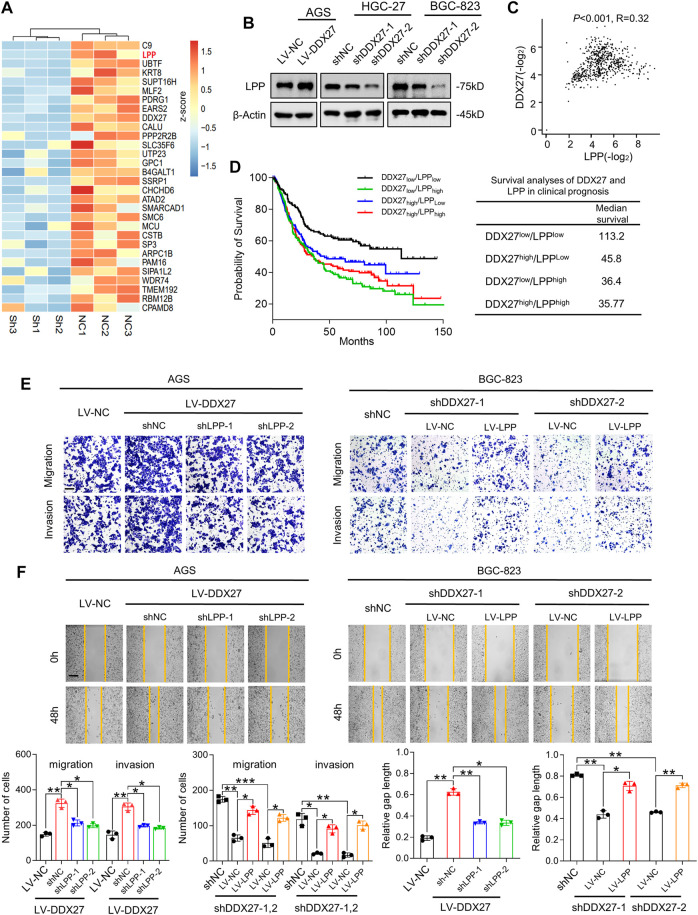
LPP is indispensable for DDX27-mediated GC migration and invasion. **(A)** Heatmap generated from mass spectrometry analyses of the protein samples isolated from HGC-27 knockdown and control cells (log_2_ fold change <−1; *p* < 0.05). LPP was identified as one of the significantly downregulated genes. **(B)** Western blot analysis of the LPP expression in DDX27 overexpressing and silencing GC cells. **(C)** Positive correlation between DDX27 and LPP was analyzed *via* TCGA and GTEx datasets of the stomach. (*p* < 0.001, *R* = 0.32, by Spearman correlation method). **(D)** Survival analysis of DDX27 and LPP in clinical prognosis (*n* = 630, left) and the median survival in different groups (right, by log-rank test). **(E)** Trans-well assays showing the migratory and invasive abilities between LPP knockdown (or overexpression) and corresponding control cells with the stable DDX27 overexpression (or knockdown). Scale bars: 12.5 μm, statistical analyses are exhibited below. (***p < 0.001, **p < 0.01, and *p < 0.05, by ANOVA). **(F)** Wound healing was performed to detect cell motility between LPP knockdown (or overexpression) and corresponding control cells with a stable DDX27 overexpression (or knockdown). Scale bars: 25 μm, statistical analyses are exhibited as follows. (**p < 0.01 and *p < 0.05, by ANOVA), *n* ≥ 3, the data are presented as the mean ± SD. LPP can be regulated by DDX27 through alternative splicing.

To illustrate the underlying mechanisms in DDX27-mediated GC metastasis, we first performed mass spectrometry (MS) between DDX27 knockdown and control cells to seek the DDX27-related potential functional proteins. MS showed that LPP was remarkably changed when DDX27 was downregulated (Figure 4A; *p* < 0.05, log_2_ fold change <−1). Considering that the DDX family has regulative potentials in alternative splicing, we performed whole-transcriptome sequencing (RNA-seq) and alternative splicing analysis. A total of 18 genes, including LPP, were identified as candidate genes by the intersection analysis of data from MS and alternative splicing analysis (Supplementary Figure S2A). Interestingly, from MS and RNA-seq, we found that the LPP protein level was significantly decreased with tiny changes of its whole transcript level after DDX27 was inhibited, which clued us that there may exist a special regulative manner between DDX27 and LPP. Based on the comprehensive results of MS, transcriptome sequencing, and alternative splicing analysis, we finally chose LPP as the DDX27 downstream target for further studies.

LPP-encoded protein localizes at the cell periphery in focal adhesions, involving in cell adhesion, cell motility, and cell-substrate cytoskeletal interactions (Jin et al., 2007; Ngan et al., 2017). Although LPP has been reported as an oncogene in various tumor malignancies (Colas et al., 2012; Leung et al., 2018), its role in GC metastasis is not fully confirmed. To better understand the role of LPP in GC, we studied its expression in the gastric cell lines and TCGA dataset. We found not only the protein level of LPP in the GC cell lines (Supplementary Figure S2B) but also the mRNA level of LPP in the GC tissues, which showed a high tendency (Supplementary Figure S2C). Kaplan–Meier analysis showed that GC patients with a high LPP expression represented a poor OS by conducting the TCGA STAD cohort analysis (Supplementary Figure S2D).

Based on the previous results, we detected the LPP expression in overexpressed or silenced DDX27 cell models. We found that LPP was obviously increased in the DDX27-overexpressed cells and was significantly inhibited when DDX27 was downregulated (Figure 4B). A positive correlation between DDX27 and LPP was observed in the TCGA gastric cancer cohort (Figure 4C). Noticeably, after dividing the GC patients into four groups according to the median expression of DDX27 and LPP, we analyzed the effects of two markers on clinical survival and found that the low expressions of DDX27 and LPP could indicate a better prognosis, while either a high DDX27 or LPP could lead to a short overall survival (Figure 4D). In the rescue assays, through silencing LPP in the DDX27 overexpressed GC cells, we found that improved metastatic ability of the GC cells caused by the DDX27 overexpression was impaired after LPP inhibition. Meanwhile, when LPP was overexpressed in the DDX27-knockdown cells, the metastatic ability of cells inhibited by DDX27 impairment was recovered (Figures 4E,F and Supplementary Figure S2F). The lentivirus efficiency of LPP is shown in Supplementary Figure S2E. We found out that LPP may be a downstream functional target of DDX27.

In addition, the single-gene GSEA using the TCGA GC cohort identified epithelial mesenchymal transition (EMT) as the main hallmarks of the LPP high expression group (Supplementary Figure S3), and we discovered the DDX27 overexpression caused EMT activation which could be alleviated by LPP inhibition, while the LPP overexpression could partly recover DDX27-induced EMT impairment (Supplementary Figure S2G). The aforementioned evidence indicated that LPP took part in DDX27-promoted metastasis by modulating the EMT process in GC cells.

### Lipoma Preferred Partner can be Regulated by DEAD-Box Helicase 27 Through Alternative Splicing

**FIGURE 5:**
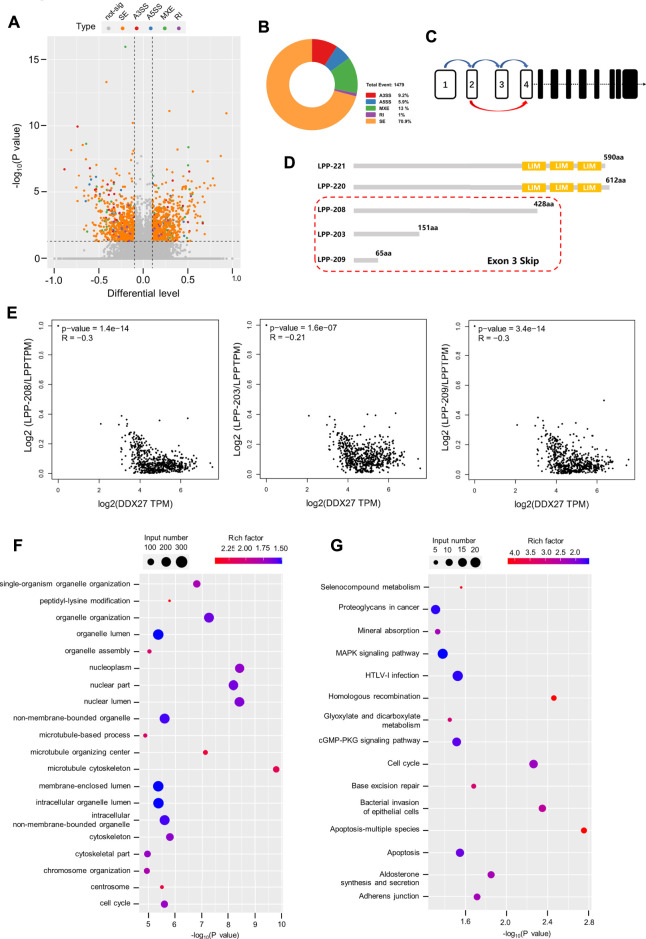
LPP can be regulated by DDX27 through alternative splicing. **(A)** Volcano plot reflects the distribution of 1,169 different alternative splicing genes. (*p* < 0.05, difference of alternative splicing events |Δψ|>0.1). **(B)** A total of 1,479 alternative splicing events were found in the alternative splicing analysis (*p* value < 0.05, |Δψ|>0.1), of which exon skipping accounted for a large proportion. **(C)** Increased exon skipping events in exon 3 of LPP in the gastric cancer cells with DDX27 knockdown. **(D)** Schematic diagram of the protein domains of several LPP transcript variants. LPP-221 and LPP-220 are the long transcript variants of LPP whose translated proteins have complete functional domains, while LPP-203, LPP-208, and LPP-209 are transcript variants of exon 3 skipped, which lacks the characteristic LIM domain. **(E)** Spearman correlation analysis results of the relative expression of LPP transcript variants and DDX27 expression. (Relative expression of LPP transcript variants was normalized with the total LPP). **(F,G)** GO/KEGG enrichment for different alternative splicing genes. The horizontal axis indicates the significance of the enrichment (expressed as −log10 (*p* value); the vertical axis indicates an enriched GO Terms/KEGG pathway (*p* value < 0.05).

Accumulative evidence revealed that the DEAD box family can participate in the cancer malignant process by regulating the alternative splicing of downstream RNAs. For example, DDX17 is reported to promote HCC metastasis by regulating the alternative splicing of PXN-AS1 (Zhou et al., 2021). However, in CRC, DDX56 is identified as a novel oncogene and prognostic biomarker that promotes alternative splicing of WEE1 (Kouyama et al., 2019). In this study, the Volcano plot data from alternative splicing analysis exhibited distribution of different alternative splicing genes in the DDX27-knockdown GC cells (Figure 5A) and showed that skipping exon (SE) was the predominant alternative splicing event after DDX27 knockdown (Figure 5B). Gene ontology (GO) and Kyoto Encyclopedia of Genes and Genomes (KEGG) pathway enrichments were performed, and the results exhibited that different splicing genes were significantly enriched in the cytoskeleton and adhesive junctions which hinted us that DDX27 may regulate cell mobility *via* alternative splicing (Figures 5F,G).

Notably, we noticed that SE events of the third exon (chr3:188225404–188225527) of the LPP full-length transcript were increased in the DDX27-knockdown cell lines (Figure 5C). LPP belongs to the LIM domain protein subfamily, characterized by a N-terminal proline-rich region and three C-terminal LIM domains (Ngan et al., 2018). After searching the ensemble database, we found that LPP has a variety of transcript variants, and none of these transcript variants with the deletion of the third exon can be translated into a relatively complete LPP protein structure containing three LIM domains, which is indispensable for the LPP protein binding to the cytoskeleton and adhesion (Figure 5D). As exhibited in Figure 5E, we found that a high expression of DDX27 was negatively correlated with the relative expression of LPP transcripts with the third exon depletion (LPP-203, LPP-208, and LPP-209) from the TCGA gastric cancer cohorts.

Taking together, we noticed that DDX27 may regulate the protein expression of LPP by reducing the skipping exon (SE) events on the third exon of LPP full-length transcripts, thereby enhancing the translation of LPP proteins with functional domains and promoting the cell motility and metastatic ability of the gastric cancer cells.

## Discussion

The DEAD box family has been suggested to act as multifunctional roles in carcinogenesis processes (Heerma van Voss et al., 2015; Heerma van Voss et al., 2017; Hashemi et al., 2019). DDX27 has also been found aberrantly overexpressed in various tumors, while its function in GC metastasis is still unknown. Here, we investigated the effects and clinical significance of DDX27 in gastric cancer and proved that DDX27 could promote the migratory and invasive ability of GC through the LPP-mediated EMT process.

For the role of DDX27 in GC, a recent study has suggested that DDX27 could regulate cell proliferation in the GC cells through the inhibition of cell cycle progression independent of apoptosis (Tsukamoto et al., 2015). Zhou et al. found that DDX27 knockdown sensitized the gastric cancer cells to epirubicin or cisplatin, and conversely its overexpression reduced the eirenicon-induced DNA damage and apoptosis (Zhou et al., 2015). They mainly focused on studying the effects of DDX27 in GC proliferation and chemotherapy. This is the first time we verified that DDX27 could also regulate GC migration and invasion. We found that in the human GC samples, DDX27 maintained a significantly upregulated expression at the mRNA and protein levels, which could also reinforce GC metastasis *in vitro* and *in vivo*. The highly expressed DDX27 indicated GC patients’ poor prognosis.

Mass spectrometry and RNA-seq analysis were conducted to screen out the DDX27 downstream candidates and found that LPP, which belongs to the zyxin family of LIM proteins, might interact with DDX27 in GC. LPP has been confirmed as a regulator of mesenchymal/fibroblast cell motility (Ngan et al., 2018). Pathologically, LPP is commonly emerged as a critical tumor inducer, accounting for tumor initiation, metastasis, and drug resistance (Ngan et al., 2013; Kuriyama et al., 2016). LPP acted as a tumor metastatic accelerator by virtue of being located inside the adhesion and promoting invadopodia formation (Kiepas et al., 2020). However, the role of LPP in GC is still not clear , so we studied the role of LPP in this study and determined that LPP was a putative target of DDX27 in GC cells.

Members of the DEAD box family of RNA helicases have been found to be involved in affecting RNA maturation, RNP assembly, and RNAs’ ultimate destiny (Linder and Jankowsky 2011). We supposed that DDX27 can also influence the alternative splicing process of LPP by influencing the RNA structure. We demonstrated our assumption with RNA-seq and alternative splicing analysis and discovered that exon 3 (chr3:188225404–188225527) skipped events of LPP transcript that happened in DDX27-knockdown cell lines. In functional rescue experiments, DDX27-conferred metastasis advantage to GC cells was abolished by LPP inhibition.

Current studies demonstrate that both DDX27 and LPP are involved in the tumor EMT process. For example, DDX27 contributes to CRC development by driving the EMT process (Tang et al., 2018). The formation of the FOXD1/CYTOR/LPP axis has been proven to be indispensable to induce EMT in oral squamous cell cancer (OSCC) (Chen et al., 2021). Here, we similarly noticed that DDX27 can regulate the EMT process in GC cells, and its effects were abrogated if LPP was knocked down, indicating that LPP is necessary in the DDX27-triggered EMT process. In addition, activation of an epithelial-mesenchymal transition (EMT) has been linked to the formation of neoplastic stem cells (Lambert and Weinberg 2021), and DDX27 has been noticed to also regulate the cancer stem cell (CSC) activity in CRC (Yang et al., 2019). There may exist a complicated interaction among DDX27, EMT, and CSCs waiting to be illuminated.

The high level of DDX27 in GC, together with its remarkable prometastasis effects, prompted that DDX27 performed a vital role in accelerating GC metastasis and might be a novel prognostic risk factor for GC patients. Our study has limitations that the detailed regulatory mechanisms of how DDX27 acts to influence the alternative splicing of LPP need more evidence to prove, and it is also the focus of our later research. In summary, we unveiled that the protumorigenic function of DDX27 was mediated through a cancer-regulating functional axis DDX27/LPP/EMT in gastric cancer.

## Data Availability

The datasets presented in this study can be found in online repositories. The names of the repository/repositories and accession number(s) can be found in the article/Supplementary Material.
