# Supplementary material for: DEAD-Box Helicase 27 Triggers Epithelial to Mesenchymal Transition by Regulating Alternative Splicing of Lipoma-Preferred Partner in Gastric Cancer Metastasis
Source: Front Genet. 2022 May 4;13:836199. doi: 10.3389/fgene.2022.836199 (PMC9114675; doi:10.3389/fgene.2022.836199)
Supplement: Supplementary file 1 [file Table1.DOCX]

**FIGURE LEGENDS**

**Figure 1. DDX27 exhibits elevated expression profile in GC. (A)** DDX27 transcript levels between adjacent non-tumor and GC tissues in GEO database (GSE13911, ****P* < 0.001, by student’s t test). **(B)** Quantitative reverse transcription PCR (qRT-PCR) (left) and western blot (right) analyses of DDX27 expression in the normal gastric epithelial cell line (GES-1) and gastric cancer cell lines (ASG, BGC-823, MKN-45, MKN-28, SNU-1, HGC-27). **(C)** DDX27 expression was analyzed by qRT-PCR in paired surgically resected GC (n=52) and adjacent non-tumor tissues (n=52) (****P* < 0.001, by student’s t test). **(D)** IHC score of DDX27 in paired clinically collected adjacent non-tumor (n=52) and GC tissues(n=52) (left, ****P* < 0.001, by student’s t test)). Representative images of staining were exhibited in the right. Scale bars: 1000μm (up) or 100μm (below). **(E)** IHC staining images of Ki-67 in adjacent non-tumor (n=52) and GC tissues (n=52). Scale bars:1000μm (up) or 100μm (below). **(F)** Correlation analysis between DDX27 and Ki-67 IHC staining score. (*P* < 0.001, R = 0.5828, by Pearson correlation method). N≥3, Data are presented as mean ± standard deviation (SD).

**Figure 2. High DDX27 expression indicated a poor clinical prognosis. (A)** Kaplan–Meier analysis of overall survival (OS) between GC patients with high (n=319) or low (n=556) DDX27 expression from dataset. (*P* < 0.001, by log-rank test). **(B, D)** IHC scoring of paired adjacent non-tumor (n=82) and GC tissues (n=98) (B, ****P* < 0.001, by student’s t-test) with representative images of IHC staining for DDX27 protein levels (D). Scale bars: 250μm (up) or 20μm (below). **(C)** Kaplan-Meier analysis was used to evaluate DDX27 expression in patients with GC form tissue microarray (*P* < 0.001, by log-rank test). N≥3, Data are presented as mean ± SD.

**Figure 3. DDX27 is a critical mediator of GC metastasis via EMT. (A, B)** Transcript level (A) and protein expression (B) of DDX27 in DDX27 knockdown and overexpression cell models (***P*< 0.01, **P* < 0.05, by ANOVA). **(C)** Metastatic ability assessed by trans-well assay for DDX27 overexpression and knockdown cell models (left, overexpression vs. control; right, knockdown vs. control). Scale bars: 12.5 μm (**P< 0.01, *P < 0.05, by ANOVA). **(D)** Wound healing and statistical analysis for indicated cells (left, overexpression vs. control; right, knockdown vs. control) Scale bars: 25 μm. (*P < 0.05, by ANOVA). **(E)** Hematoxylin-eosin (H&E) staining for metastatic nodules in dissected lung specimen from nude mice (10 mice in each group). Scale bars:1000 μm (up) or 100 μm (below). **(F)** Protein level of EMT markers (E-cadherin, N-cadherin, Vimentin, ZO-1) among indicated GC cells. N≥3, Data are presented as mean ± SD.

**Figure 4. LPP is indispensable for DDX27-mediated GC migration and invasion.** **(A)** Heatmap generated from mass spectrometry analyses of protein samples isolated from HGC-27 knockdown and control cells (log_2_ fold change＜-1; P < 0.05). LPP was identified as one of the significantly downregulated genes. **(B)** Western blot analysis of LPP expression in DDX27 overexpressing and silencing GC cells. **(C)** Positive correlation between DDX27 and LPP was analyzed via TCGA, GTEx datasets of stomach. (P<0.001, R=0.32, by Spearman correlation method). **(D)** Survival analysis of DDX27 and LPP in clinical prognosis (n=630, left) and the median survival in different groups (right, by log-rank test). (**E**) Trans-well assays showing migratory and invasive abilities between LPP knockdown (or overexpression) and corresponding control cells with stable DDX27 overexpression (or knockdown). Scale bars: 12.5μm, statistical analyses are exhibited below. (****P* < 0.001, ***P* < 0.01, * *P* < 0.05, by ANOVA). (**F**) Wound healing was performed to detect cell motility between LPP knockdown (or overexpression) and corresponding control cells with stable DDX27 overexpression (or knockdown). Scale bars: 25μm, statistical analyses are exhibited below. (***P* < 0.01, * *P* < 0.05, by ANOVA), N≥3, Data are presented as mean ± SD.

**Figure 5. LPP can be regulated by DDX27 through alternative splicing.** **(A)** Volcano plot reflects distribution of 1169 different alternative splicing genes. (*P*<0.05, difference of alternative splicing events |Δψ|>0.1). **(B)** A total of 1479 alternative splicing events were found in the alternative splicing analysis (P value<0.05, |Δψ|>0.1), of which exon skipping accounted for a large proportion. **(C)** Increased exon skipping events in exon 3 of LPP in gastric cancer cells with DDX27 knockdown. **(D)** Schematic diagram of the protein domains of several LPP transcript variants. LPP-221 and LPP-220 are long transcript variants of LPP whose translated proteins have complete functional domains, while LPP-203, LPP-208, and LPP-209 are transcript variants of exon 3 skipped, which lacks the characteristic LIM domain. **(E)** Spearman correlation analysis results of relative expression of LPP transcript variants and DDX27 expression. (Relative expression of LPP transcript variants were normalized with total LPP). **(F, G)** GO/KEGG enrichment for different alternative splicing genes. The horizontal axis indicates the significance of the enrichment (expressed as -log10 (P value), the vertical axis indicates enriched GO Terms/KEGG pathway (P Value<0.05).

**SUPPLEMENTARY FIGURE LEGENDS**

**Figure S1 (A)** Box and whisker plot showing DDX27 transcript levels between tumor tissues (T) and normal tissues (N) in different kinds of gastrointestinal cancers analyzed by TCGA dataset (analyzed by ANOVA, *Q＜0.01). **(B)** Clinical characteristics of DDX27 in 92 patients in gastric cancer cohort (OncoSG, 2018). **(C)** Migratory and invasive ability assessed by trans-well (scale bars: 12.5 μm) and wound healing assay (scale bars: 25 μm) for DDX27 knockdown HGC-27 cells (**P < 0.01, *P < 0.05, by ANOVA). **(D)** Statistical analysis for metastatic nodules in dissected lung specimen from nude mice (10 mice in each group, by ANOVA). **(E)** Enrichment plot of epithelial-mesenchymal transition (EMT) hallmarks by Gene Set Enrichment Analysis (GSEA) comparing DDX27-deficient GC cells to controls. NES (normalized enrichment score), **p < 0.01, * p < 0.05，*** p < 0.001, N≥3, Data are presented as mean ± standard deviation (SD).

**Figure S2 LPP is indispensable for DDX27-mediated GC migration and invasion. (A)** Intersection analysis between mass spectrometry (MS) and alternative splicing analysis. 18 candidates were found after overlapping. **(B)** Western blot analyses of LPP expression in the normal gastric epithelial cell line (GES-1) and gastric cancer cell lines (HGC-27, ASG, BGC-823, MKN-45). **(C)** Box and whisker plot showing LPP transcript levels between normal tissues (NT) and GC tissues (GC) analyzed from TCGA dataset by student’s t-test. **(D)** Kaplan-Meier analysis was used to evaluate the clinical prognosis of LPP expression in patients with GC by log-rank test. Elevated LPP expression indicated poor overall survival. **(E)** Protein expression of LPP for LPP knockdown and overexpression of DDX27 overexpressing and silencing cells. **(F)** Trans-well (scale bars: 12.5 μm) and wound healing assay (scale bars: 25 μm) were performed to detect cell motility between LPP overexpression and control cells with stable DDX27 knockdown. statistical analyses are exhibited below. (**P < 0.01, *P < 0.05, by ANOVA). **(G)** Protein level of EMT markers (E-cadherin, N-cadherin, Vimentin, ZO-1) among LPP knockdown (or overexpression) cell models of DDX27 overexpressing (or silencing) cells, N≥3, Data are presented as mean ± SD.

**Figure S3** Enrichment plot of top 6 scored hallmarks by Single-gene GSEA analysis of LPP high expression group vs. LPP low expression group.
