# Supplementary figures and images for: DEAD-Box Helicase 27 Triggers Epithelial to Mesenchymal Transition by Regulating Alternative Splicing of Lipoma-Preferred Partner in Gastric Cancer Metastasis
Source: Front Genet. 2022 May 4;13:836199. doi: 10.3389/fgene.2022.836199 (PMC9114675; doi:10.3389/fgene.2022.836199)

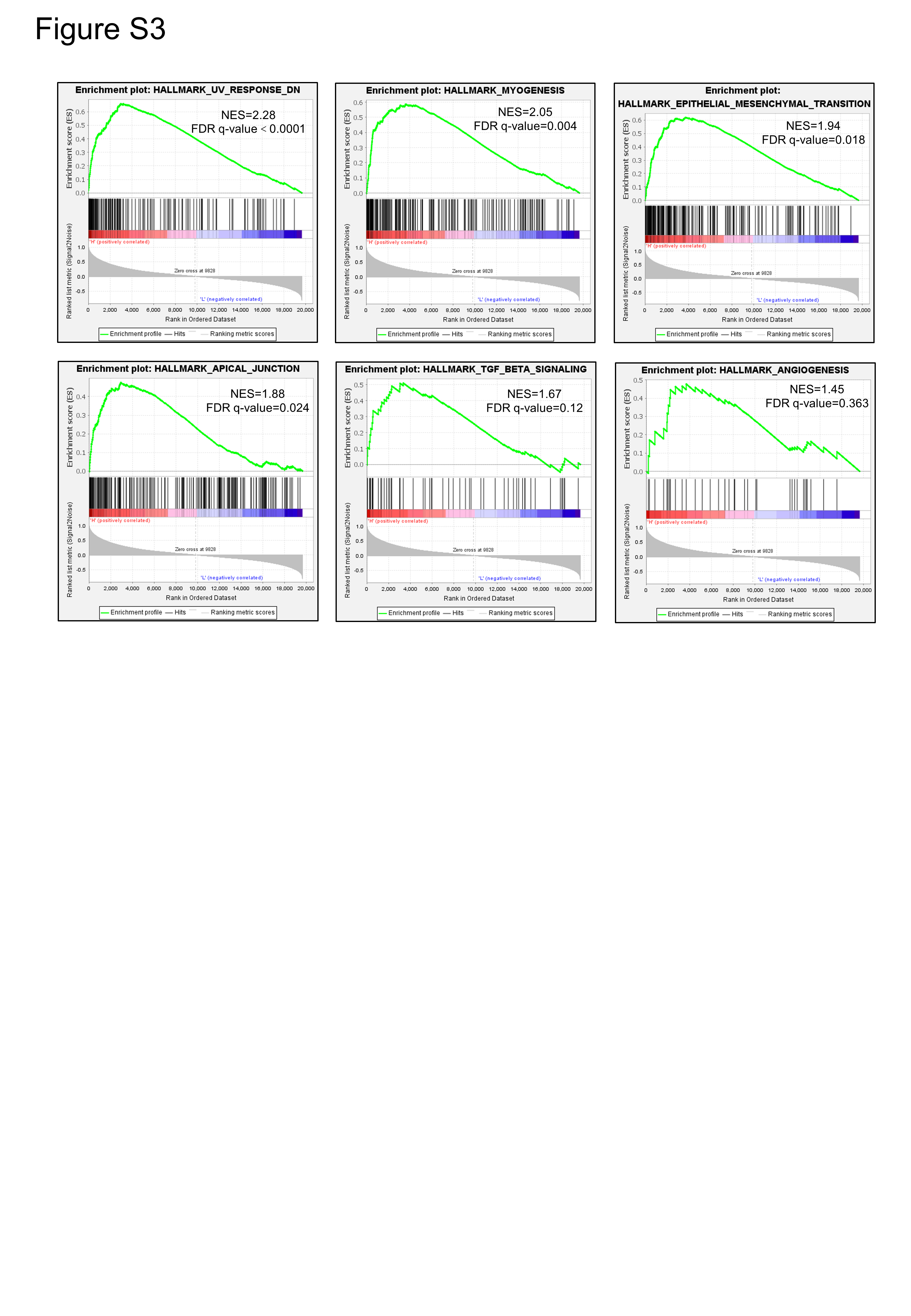

Supplement: Supplementary file 2 [file Image3.TIF]

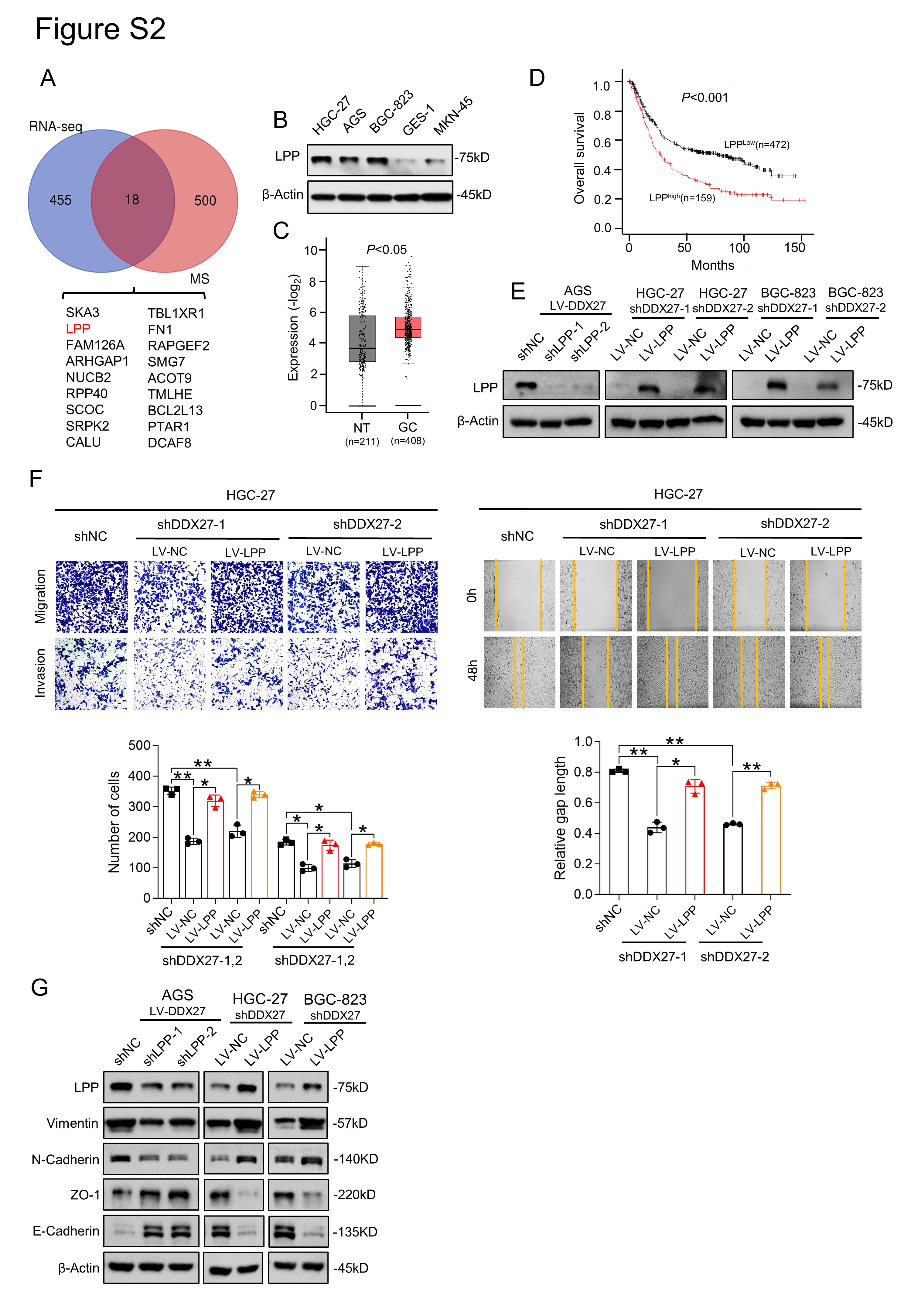

Supplement: Supplementary file 3 [file Image2.TIF]

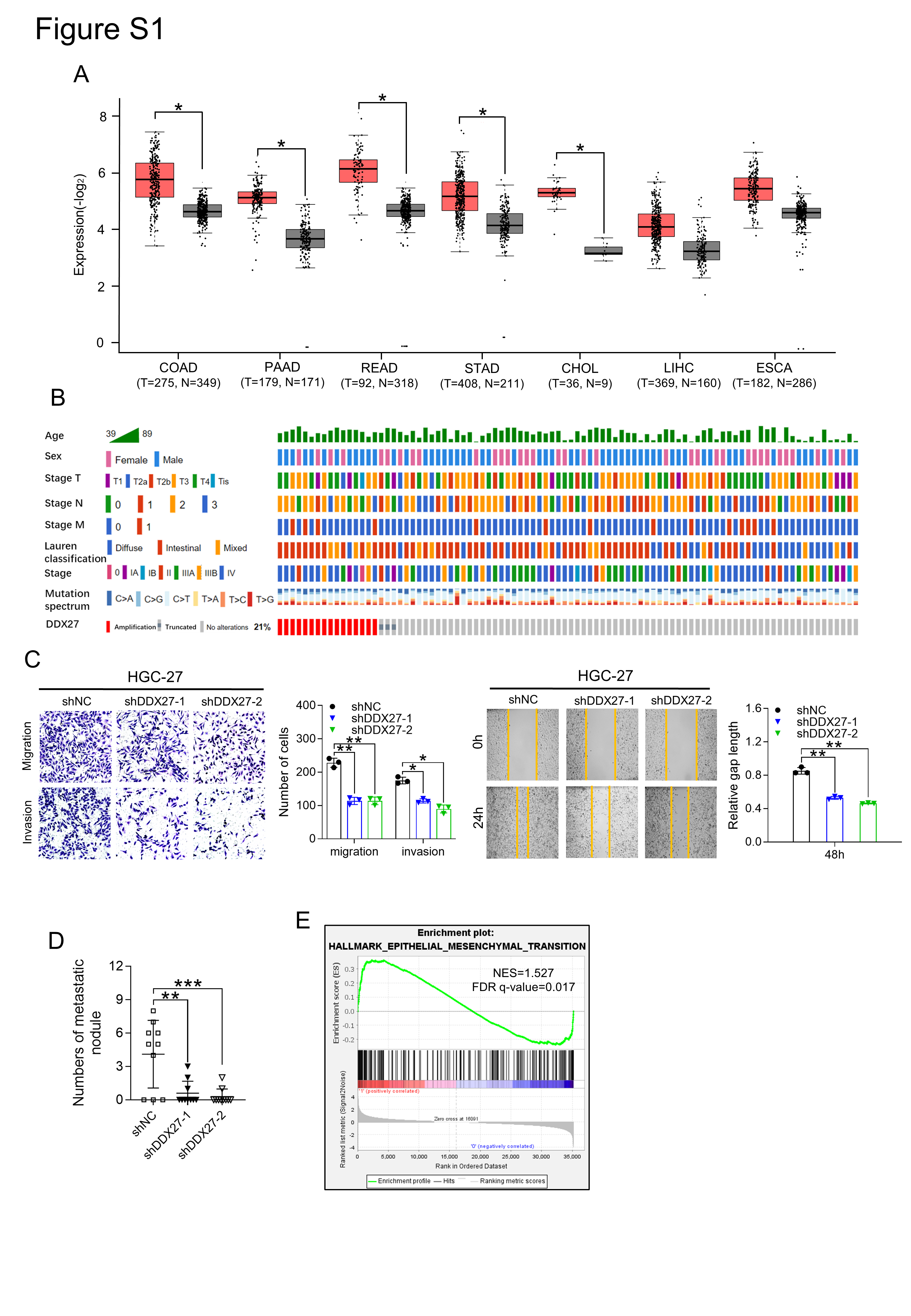

Supplement: Supplementary file 4 [file Image1.TIF]
