# Supplementary material for: DEAD-Box Helicase 27 Triggers Epithelial to Mesenchymal Transition by Regulating Alternative Splicing of Lipoma-Preferred Partner in Gastric Cancer Metastasis
Source: Front Genet. 2022 May 4;13:836199. doi: 10.3389/fgene.2022.836199 (PMC9114675; doi:10.3389/fgene.2022.836199)
Supplement: Supplementary file 5 [file Table5.DOCX]

**Supplementary table 1. Sequences of primers or targets for qRT-PCR and shRNA**

| Gene | | sequence | | Method |
| --- | --- | --- | --- | --- |
| DDX27 | 5’-AGCCCGTGGACTTGACATTG-3’ | | qRT-PCR (Forward) | |
| DDX27 | 5’-GCATCTTCCGCTCATCTTCTC-3’ | | qRT-PCR (Reverse) | |
| LPP | 5’-GTTTCACCTGCGTGATGTGCCA-3’ | | qRT-PCR (Forward) | |
| LPP | 5’-GGCTGGCATAATAGGCTCCTTG-3’ | | qRT-PCR (Reverse) | |
| β-actin | 5’-GGTCATCACCATTGGCAA-3’ | | qRT-PCR (Forward) | |
| β-actin | 5’-GAGTTGAAGGTAGTTTCGTGGA-3’ | | qRT-PCR (Reverse) | |
| DDX27 | 5’-AGGAATTTGACTTGGCCTT-3’ | | DDX27-RNAi-1 | |
| DDX27 | 5’-GATCCAGAAGGCGTGCATA-3’ | | DDX27-RNAi-2 | |
| DDX27 | 5’-TCAAATTCCGGGACAAGAT-3’ | | DDX27-RNAi-3 | |
| LPP | 5’- CCCAACCAGGGACGCTATTAT-3’ | | shRNA-LPP-1 | |
| LPP | 5’- CGCTATTATGAAGGCTACTAT-3’ | | shRNA-LPP-2 | |
| LPP | 5’- GAAATCTCCATCCTATCATTT-3’ | | shRNA-LPP-3 | |
|  |  | | |  |
