# Supplementary material for: DEAD-Box Helicase 27 Triggers Epithelial to Mesenchymal Transition by Regulating Alternative Splicing of Lipoma-Preferred Partner in Gastric Cancer Metastasis
Source: Front Genet. 2022 May 4;13:836199. doi: 10.3389/fgene.2022.836199 (PMC9114675; doi:10.3389/fgene.2022.836199)
Supplement: Supplementary file 6 [file Table6.DOCX]

**Supplementary table 2. the details about datasets used in the study**

| **Public Database** | **Datasets** | **Gene/Probe ID** | **Source** |
| --- | --- | --- | --- |
| Gene Expression Omnibus (GEO)  (https://www.ncbi.nlm.nih.gov/geo/) | GSE13911 | 215693_x_at  (DDX27) | Figure 1A |
| Kaplan-Meier Plotter  (http://kmplot.com/analysis/) | GSE14210; GSE15459  GSE22377; GSE29272  GSE38749; GSE51105  GSE62254 | 221780_s_at  (DDX27) | Figure 2A |
| GEPIA2  (http://gepia2.cancer-pku.cn/) | TCGA STAD  (Tumor and Normal)  GTEx (stomach) | DDX27  LPP | Figure 2C |
| Kaplan-Meier Plotter  (http://kmplot.com/analysis/) | GSE14210; GSE15459  GSE22377; GSE29272  GSE38749; GSE51105  GSE62254 | 221780_s_at  (DDX27)  224811_at  (LPP) | Figure 4D |
| GEPIA2  (http://gepia2.cancer-pku.cn/) | TCGA STAD  (Tumor and Normal)  GTEx (stomach) | DDX27  LPP  LPP-208  LPP-203  LPP-209 | Figure 5E |
| GEPIA2  (http://gepia2.cancer-pku.cn/) | TCGA COAD  TCGA PAAD  TCGA READ  TCGA STAD  TCGA CHOL  TCGA LIHC  TCGA ESCA | DDX27 | Figure S1A |
| cBioPortal  (https://www.cbioportal.org/) | OncoSG, 2018 | DDX27 | Figure S1B |
| Kaplan-Meier Plotter  (http://kmplot.com/analysis/) | GSE14210; GSE15459  GSE22377; GSE29272  GSE38749; GSE51105  GSE62254 | 224811_at  (LPP) | Figure S2D |
| GEPIA2  (http://gepia2.cancer-pku.cn/) | TCGA STAD | LPP | Figure S2C |
